# The evolution of chemical defenses along invasion routes: *Harmonia axyridis* Pallas (Coccinellidae: Coleoptera) as a case study

**DOI:** 10.1002/ece3.4299

**Published:** 2018-07-24

**Authors:** Alexandra Magro, Felipe Ramon‐Portugal, Benoît Facon, Christine Ducamp, Jean‐Louis Hemptinne

**Affiliations:** ^1^ UMR CNRS EDB Université Paul Sabatier Toulouse France; ^2^ ENSFEA Castanet‐Tolosan France; ^3^ UMR INRA PVBMT CIRAD Saint Pierre‐La Réunion France

**Keywords:** alkaloids, evolution of increased competitive ability, harmonine, hydrocarbons, immunity, ladybirds

## Abstract

The evolution of increased competitive ability (EICA) hypothesis (Blossey & Nötzold, 1995) postulates that escaping from coevolved enemies increases invaders fitness by energy reallocation from defenses and immunity to growth and reproduction. In this context, we evaluated the evidence of evolutionary change in invasive populations of *Harmonia axyridis* Pallas (Coccinellidae: Coleoptera). We measured egg defenses—cocktail of hydrocarbons on the egg's surface flagging egg toxicity and the concentration of the main alkaloid harmonine—in individuals from three populations along the invasion route (Japan: native, United States: introduced more than 30 years ago, South Africa: introduced in the early 2000s) in a common garden experiment. Our results support the EICA hypothesis: We found changes along the invasion route in the profiles of the hydrocarbons coating the eggs’ surface and a decrease in the concentration of harmonine in eggs from the most recent invasive South African population compared to the long established in the United States and the native Japanese ones.

## 1 INTRODUCTION

The number of introduced species rocketed in the 20th Century as trade, fueled by fossil energies, went global. As a consequence, the number of conservation and economic problems caused by many non‐native species increased drastically and led the scientific community to produce an astonishing amount of work on biological invasions (Lockwood, Hoopes, & Marchetti, 2007). Nevertheless, the mechanisms that turn an alien species invasive are still little understood (Lee & Klasing, 2004), although a clear understanding is essential for limiting the effects of invasive alien species.

The vast majority of empirical studies on invaders have been conducted on primary producers (75%) and a small number on herbivores (10%) while research on invasive predators and parasites lags far behind (Lowry et al., 2013). In this work, we focused on the Harlequin ladybird, *Harmonia axyridis* Pallas (Coccinellidae: Coleoptera), a generalist predator of aphids (Aphididae: Hemiptera). Native to Asia (Iablokoff‐Khnzorian, 1982), it was first used as a biocontrol agent (Roy et al., 2016) before recently becoming fairly abundant and rapidly invading four different continents (Hemptinne, Magro, Evans, & Dixon, 2012; Lombaert et al., 2010). Roy, Brown, and Majerus (2006) and Roy and Wajnberg (2008) claim this predator to be “the most invasive ladybird on Earth” and consider that it could serve as an important model for studying invasive alien species. In addition, its effects on native ladybird species (Roy et al., 2012; Honek et al., 2016) but also on human health and well‐being (Sloggett et al., 2011) call for further research efforts to unravel the *H. axyridis* invasion process.

Leifso et al. (2012) state that the expansion of a species’ range boundaries, from the point of view of the invader, can be driven by mechanisms that are either trait‐based or circumstantial (e.g. propagule pressure). This expansion can also derive from the recipient community's traits and environmental context. In the case of *H. axyridis*, most of the work has focused on trait metrics that may confer an advantage in relation to native species. However, the invader's evolutionary changes, which could be crucial to understanding what exactly facilitated invasion, have hardly been studied at all (Colautti & Lau, 2015; Sloggett, 2012). Yet, Lombaert et al. (2010) genetic study of *H. axyridis* invasive populations suggests that an evolutionary shift might have facilitated invasion, but the exact instrumental phenotypic adaptations are still under debate (Sloggett, 2012). In the recent past, the review work by Roy et al. (2016) confirmed the paucity of studies on the traits of both native and alien populations of *H. axyridis* and recommended they should be extended and combined to provide insights into patterns and evolutionary processes at the origin of the invasion. This study explores some of these processes, namely the evolution of chemical defenses.

Rieder, Newbold, Sato, Yasuda, and Evans (2008) provided one of the rare comparative analyses *of the H. axyridis* populations. The authors studied the intraguild predation of the Japanese *Coccinella septempunctata* L. larvae on *H. axyridis* eggs both from the Japanese and North American populations. Their results indicate that the eggs from the Japanese (i.e. native) *H. axyridis* population are better defended than those from the North American (i.e. invasive) population. *C. septempunctata* is more reluctant to eat Japanese eggs than North American eggs and, when constrained to do so, suffers from a higher mortality or an extended developmental time than when preying upon North American eggs. These dissimilarities in egg defenses in native and invasive populations of *H. axyridis* could be due to enemy release (Rieder et al., 2008), which has been suggested as contributing to its invasive success (Roy, Lawson Handley, Schönrogge, Poland, & Purse, 2011). Indeed, the natural enemies of ladybird beetles are well documented, and include a diversity of invertebrate and vertebrate predators and parasites as well as pathogens, some being widely distributed while others have more limited ranges (Ceryngier, Roy, & Poland, 2012). However, there is a lack of comprehensive studies assessing the natural enemies of *H. axyridis* in its native and introduced range (Haelewaters et al., 2017; Roy, Rhule, et al., 2011). Nevertheless, Ceryngier et al. (2012) point out that natural enemies, namely parasitoids, are more diverse in the invader's native range, although the acquisition of new parasitoids might in the long term, lead to novel associations in the invaded areas (Ceryngier et al., 2018).

Enemy release can confer other advantages to the invader than just a lower mortality rate. As far as invasive plants are concerned, research programs have focused on testing the evolution of increased competitive ability (EICA) hypothesis (Atwood & Meyerson, 2011). This hypothesis posits that in the absence of specialized herbivores in alien environments, selection will favor plant genotypes with modified trade‐offs reducing resource allocation to herbivore defense and consequently enhancing their competitive abilities (Blossey & Nötzold, 1995). To an undeniable degree, resource investment in defense is costly to plant growth and reproduction (Herms & Mattson, 1992), and this is certainly true for animals too (Braendle, Heyland, & Flatt, 2011). Regarding insects, investment in egg defense is high as these immature stages are immobile and most often devoid of parental attendance (Hilker & Meiners, 2002). In the case of ladybirds, eggs are protected internally by de novo synthetized alkaloids conferring egg toxicity (King & Meinwald, 1996) and externally by a cocktail of hydrocarbons coating the eggs’ surface and known to flag internal egg toxicity (Hemptinne, Lognay, Gauthier, & Dixon, 2000). Therefore, on the basis of the results of the behavioral experiments of Rieder et al. (2008) on differential chemical defenses, and those of Facon et al. (2011) and Laugier et al. (2013) showing that generation time, lifetime performance and reproductive success were improved in invasive compared to native populations of *H. axyridis*, we hypothesize that EICA might be at work in the invasion process of the Harlequin ladybird.

In this study, we tested the predictions of the EICA hypothesis on the *H. axyridis* invasion by performing a common garden experiment to compare the defenses of individuals from three different populations along the invasion route (according to Lombaert et al., 2010): Japan (native population), United States of America (introduced in the 80s from Asia), and South Africa (presence first reported in 2004, introduced from the United States). This study was carried out in collaboration and in parallel to the life‐history trait measurements performed by Facon et al. (2011) and using the same biological material. Common garden experiments are classically used to empirically test EICA (Asplen et al., 2012) because they allow direct comparisons of phenotypic differences between native and invasive genotypes while minimizing the effects of phenotypic plasticity in the examination of evolved trait differentiation (Cornet, Brouat, Diagne, & Charbonnet, 2016). Rieder et al.'s (2008) work indicates that studying differences in egg defenses implies taking two aspects into consideration: Contrasting feeding deterrence should be related to information conferred by the cocktail of hydrocarbons on the eggs’ surface (egg coating hydrocarbons —ECHCs); differential mortality points to differences in egg toxicity itself, that is, in the concentration of the main alkaloid, the long chain diamine harmonine ((17R,9Z)‐1,17‐diaminooctadec‐9‐ene) (Sloggett et al., 2011). Along the invasion route, we can therefore expect changes in the ECHCs profiles and in the overall quantity of ECHCs and a decrease in the concentration of harmonine in the eggs.

## 2 MATERIAL AND METHODS

### 2.1 Ladybird cultures

Around one hundred individuals from three populations—Japan (Kyoto), United State (Brookings—Dakota), and South Africa (Bethlehem)—were captured in the field in 2008, and stock cultures were kept in the laboratory (see Facon et al., 2011).

Before the experiments, individuals were reared at constant environmental conditions (23°C; 65% RH; LD 14:10) to avoid potential biases due to maternal effects. For two generations, they were fed with ionized *Ephestia kuehniella* (Lepidoptera: Pyralidae) eggs. At generation G2, males and females were separated immediately after emergence to prevent mating. They were then maintained in the same environmental conditions for 2 weeks to ensure that all the individuals had reached reproductive maturity. From then on, the adults were reared for the chemical ecology experiments as described below.

### 2.2 Production of eggs for chemical ecology experiments

The adults from the three populations were reared at 21 ± 1°C and LD 16:8 in 2,000 cm^3^ plastic boxes containing a piece of corrugated paper. Three times a week they were fed an excess of pea aphids *Acyrthosiphon pisum* Harris, a cosmopolitan *H. axyridis* prey species. A stem of broad bean *Vicia faba* L. (“Primabel” variety) was added to each box to improve the survival of the aphids. Each rearing day, the eggs were collected and placed in 175 cm^3^ plastic boxes. Once the eggs hatched, the larvae were kept in 175 cm^3^ plastic boxes and reared in batches of 10 individuals per box, as described above. The adults from the three populations were collected from the stock cultures when they emerged from the pupae and each individual was isolated in a 50‐mm Petri dish and fed an excess of pea aphids in similar temperature and photoperiod conditions as the stock cultures. Three days later, when the cuticle hardened, the males and the females were identified. Five to six pairs were formed per population, and each pair was placed in a 90‐mm Petri dish, with a piece of corrugated paper, an excess of aphids, and a broad bean stem. They were transferred to new Petri dishes three times a week until they were 15 days old. We then started changing the Petri dishes daily and collecting the eggs laid on the filter paper during the previous 24 hr. After wetting the filter paper with a drop of de‐ionized water to dissolve the “glue” that attaches the eggs to the oviposition substrate, the eggs were detached from the support using the round tip of a closed Pasteur glass pipette.

### 2.3 Extraction of ECHCs

Every day the eggs collected from each pair were placed in a 5‐mL hemolyse tube and washed in 1 ml of *n*‐hexane for liquid chromatography (Merck) for 2 minutes, without shaking to avoid breaking the eggs. The solvent was then transferred into a 1.5‐ml chromatography vial while the eggs that remained in the tube were observed under a dissecting microscope: Whenever an egg was damaged, the whole extraction sample was discarded to avoid contamination of the egg coating with molecules from inside the eggs. The extracts were stocked at 4°C, until the GC‐MS analysis was performed (see below). Forty‐eight to 50 washed eggs per population were weighted at 0.1 μg on a Sartorius Microbalance—SC2 and conserved at −20°C for posterior analyses of the internal content of alkaloids (see below). For each population, we analyzed three batches of 330 to 360 eggs.

### 2.4 Extraction of harmonine from the eggs

Following the extraction of the ECHCs, 48 to 50 eggs per population were analyzed for harmonine content. As detection of harmonine in the GC‐MS requires derivatization, we followed the method described by Attygalle (1998): Each egg was introduced into a glass tube (12 mm diameter, 75 mm length) with 100 μl of C30 at 12,5 ng/μl in hexane and was crushed using a Pasteur pipette closed tip and left for 1 hr during which the tube was lightly shaken twice (at 30 m and 1 hr); 10 μl of MBTFA (*N*‐methyl‐bis(trifluoroacetamide)) was then added to the tube, and the mixture was heated at 80°C for 1 hr. The reaction mixture containing the resulting harmonine bis‐trifluoroacetyl derivative was stocked at −20°C pending the GC‐MS analysis.

### 2.5 Chemical analyses of the ECHCs

The GC‐MS analyses were performed on a GC‐MS TSQ Quantum chromatograph (Thermo Scientific, Illkirch, France) directly coupled to a mass spectrometer quadrupole detector (electron impact at 70 eV). The temperature source was set at 200°C, the interface between the GC and MS modules at 250°C, and the splitless injector at 280°C. The carrier gas was helium and the flow rate 1.2 ml/min. Samples of 1 μl were injected with an autosampler into an apolar capillary column (ZB‐5‐MSi 30 m × 0.25 mm, 0.25 μm film thickness, 5% diphenyl and 95% dimethylpolysiloxane). The chromatograph oven was programmed as follows: 50°C for 1 min, then increased from 50 to 140°C by 20°C per min, from 140 to 300°C by 3°C per min, and finally maintained at 300°C for 3 min. The mass spectra were scanned in full scan mode from 60 to 500 *m*/*z* values. The whole system was controlled by Xcalibur data software, version 2.1.

The identification of the hydrocarbons’ structure was determined using the mass spectral fragmentation patterns, comparison of the retention times with those of injected known compounds and the NIST library spectra. A standard mixture of alkanes, from n‐C12 to n‐C60 (Supelco, Sigma‐Aldrich, 0.01% w/w each component), was used as a qualitative reference. The nonadecane (Sigma‐Aldrich) was used as the internal standard. In order to quantify each compound, 5 μl of extract was mixed with 5 μl of a nonadecane solution at 0.0781 mg per liter in *n*‐hexane.

We used the International Union of Pure and Applied Chemistry (IUPAC) nomenclature. This nomenclature uses a descriptor (XX) for the total number of carbons in the hydrocarbon component (C_XX_; i.e. nonacosane becomes n‐C_29_), the number of double bonds (Y) follows a colon (C_XX:Y_; i.e. heneicos‐6‐ene becomes C_21:6_), and the location of methyl groups uses the descriptor (X‐Me; i.e. 3‐methylheptacosane becomes 3‐MeC_27_ and 7,12‐dimethyloctacosane becomes 7,12‐diMeC_28_).

### 2.6 Chemical analyses of the harmonine in the eggs

The harmonine was analyzed using GC‐MS as described above, but the temperature profile and the detection mode were as follows: The chromatograph oven was programmed at 50°C for 1 min, then increased from 50 to 300°C by 10°C per min, and finally maintained at 300°C for 5 min. The mass spectra were scanned from 60 to 500 *m*/*z*. The whole system was controlled using an Xcalibur data system, version 2.1.

Acquisition was performed using single‐ion‐monitoring (SIM). The identification ions were 140, 361, and 405 *m*/*z* for harmonine and from 71, 85, and 99 *m*/*z* for nC30 used for quantification (μg/mg of egg).

### 2.7 Statistical analyses

#### 2.7.1 Comparison of the ECHCs

The average mass of the eggs was calculated on samples of 48 to 50 eggs for each of the three populations. They were compared using an ANOVA after the requirements for homoscedasticity and normality had been checked for. The total quantity of hydrocarbons on the chorion of the eggs from the three populations was corrected for egg mass differences before being compared using a Kruskal–Wallis test. There were three replicates per population.

Nonmetric multidimensional scaling ordination analysis (nMDS) was used to assess overall similarities among the ECHCs profiles. This method is recommended when the number of variables exceeds the number of observations (Anderson, 2006). The nMDS was based on Jaccard similarity, and the data were transformed into presence (1) or absence (0) prior to analysis. We used the “metaMDS” function of the “VEGAN” package (Oksanen et al., 2008) in the R statistical software. In addition, based on the ECHCs profiles, the multivariate dispersion was measured for each population using the betadisper function of the Vegan R package.

#### 2.7.2 Comparison of harmonine concentration in the eggs

The concentration of harmonine in the eggs laid by the ladybirds of the three populations was analyzed using a GLM with a Poisson family. The three populations were compared using a priori orthogonal contrasts.

All the analyses were performed using R Commander, R statistical software version 2.14.1 (R Development Core Team, 2008).

## 3 RESULTS

The results of the qualitative and quantitative analyses of the ECHCs for the three populations are presented in Table 1. A total of 65 compounds were detected: 17 linear alkanes, 10 monomethylalkanes, two di‐methyl alkanes, 10 alkenes, two decyl acetates, and 24 unidentified compounds. The identification of the alkenes needs to be confirmed using derivatization (see Francis & Veland, 1981 and Vicenti, Guiglielmetti, Cassani, & Tonini, 1987 for details). The number of ECHCs decreases slightly along the invasion route, with 59 molecules for Japan, 56 for the United States, and 55 for South Africa. The mass of the eggs laid by the ladybirds of the three populations differs significantly (*F* = 12.25; 2 and 143 *df*;* p* < 0.00001), with ladybirds of the two invasive populations laying significantly larger eggs than the native Japanese (comparison Japan–South Africa: *t* = 5.7960; *p* < 0.00001; comparison Japan–United States: *t* = 5.8820; *p* < 0.00001; comparison United States–South Africa: *t* = 0.0850; *p* = 0.9960). Neither the total amount of ECHCs per egg or per μg of egg differ significantly between populations (per egg: χ^2^ = 0.0889; 0, 2 *df*;* p* = 0.9565; per ug of egg: χ^2^ = 0.80; 2 *df*;* p* = 0.6703).

**Table 1:** Results of the qualitative and quantitative (pg per egg) analysis of the compounds present on the egg coating of three populations—South Africa (SA), United States of America (USA), and Japan (JAP)—of *Harmonia axyridis* (each the mean of three trials)

Compound	KI	SA	USA	JAP
		Mean (SD)	Mean (SD)	Mean (SD)
nC14	1,400	117 (102)	61 (106)	61 (106)
nC15	1,500	55 (95)	42 (73)	53 (92)
Ni	1,518			36 (62)
Ni	1,542			32 (56)
3‐MeC15	1,565			24 (41)
nC16	1,600	341 (171)	271 (103)	319 (183)
6‐MeC16	1,639	34 (58)		29 (50)
5‐MeC16	1,685	70 (66)	76 (72)	54 (93)
nC17	1,700	347 (186)	320 (40)	199 (228)
3‐MeC17	1,730	144 (59)	171 (47)	118 (108)
Ni	1,774	35 (61)		21 (36)
nC18	1,800	405 (157)	394 (122)	326 (52)
Tetradecyl acetate	1,875	94 (87)	88 (88)	
C19:1	1,884	102 (32)	80 (78)	22 (38)
Ni	1,898	185 (61)	210 (84)	196 (116)
Ni	1,935	31 (54)		
7‐MeC19	1,941	261 (67)	211 (70)	209 (92)
5‐MeC19	1,959		27 (47)	
C20:1	1,977	473 (134)	357 (222)	145 (52)
Ni	1,982			21 (37)
Ni	1,989	39 (68)		31 (53)
nC20	2,000	187 (32)	146 (73)	155 (26)
Hexadecyl acetate	2,015	99 (18)	23 (39)	23 (39)
NI	2,022	131 (71)	52 (47)	135 (159)
C21 :2	2,031	27 (47)	32 (56)	22 (37)
C21 :1	2,078	219 (75)	240 (107)	85 (80)
Ni	2,087	552 (150)	495 (355)	163 (188)
nC21	2,100	133 (62)	107 (39)	90 (4)
5‐MeC21	2,105	66 (61)	55 (48)	72 (68)
Ni	2,113	210 (149)	126 (74)	113 (26)
Ni	2,116		19 (29)	23 (39)
Ni	2,124	983 (182)	858 (326)	806 (346)
Ni	2,132	393 (158)	286 (104)	254 (122)
5,8‐diMeC20	2,149	150 (18)	94 (85)	119 (39)
6,7‐diMeC20	2,166	68 (117)	49 (42)	37 (63)
C22:1	2,178	1,141 (364)	1,316 (712)	579 (81)
Ni	2,190	30 (52)	14 (25)	
nC22	2,200	416 (125)	317 (88)	320 (35)
Ni	2,216	2,568 (679)	2,825 (753)	2,031 (537)
Ni	2,225	216 (29)	196 (114)	157 (59)
Ni	2,235	302 (71)	294 (146)	260 (77)
C23:1	2,279	274 (75)	397 (270)	149 (83)
nC23	2,300	1,911 (2,832)	3,854 (726)	3,948 (678)
Ni	2,309		46 (79)	191 (168)
Ni	2,317	944 (325)	991 (424)	693 (111)
6‐MeC23	2,336	186 (41)	212 (81)	178 (70)
nC24	2,400	305 (39)	261 (36)	311 (100)
Ni	2,427		67 (59)	
Ni	2,430		28 (49)	
C25:1	2,475	2,645 (851)	2,168 (337)	1,868 (227)
C25:1	2,482	29 (50)		24 (41)
nC25	2,500	1,136 (266)	947 (138)	979 (186)
Ni	2,541	59 (102)	185 (60)	50 (44)
Ni	2,549		35 (60)	495 (857)
nC26	2,600	226 (55)	239 (49)	314 (190)
C27:1	2,676	1,783 (483)	1,492 (177)	1,378 (199)
nC27	2,700	473 (141)	483 (60)	540 (270)
5‐MeC27	2,749	282 (47)	217 (42)	274 (32)
nC28	2,800	160 (84)	243 (90)	283 (250)
C29:1	2,877	415 (80)	382 (64)	484 (122)
nC29	2,900	248 (129)	299 (105)	374 (220)
nC30	3,000	103 (116)	126 (136)	131 (228)
Ni	3,004	212 (46)	290 (61)	374 (222)
C31:1	3,078	516 (128)	450 (72)	319 (296)
nC31	3,100	59 (102)	96 (95)	128 (137)
Total		22685 (7,660)	23394 (4,820)	20,840 (4,660)

*Note*

KI: Kovats retention index; NI: unidentified compound.

**Figure 1:**
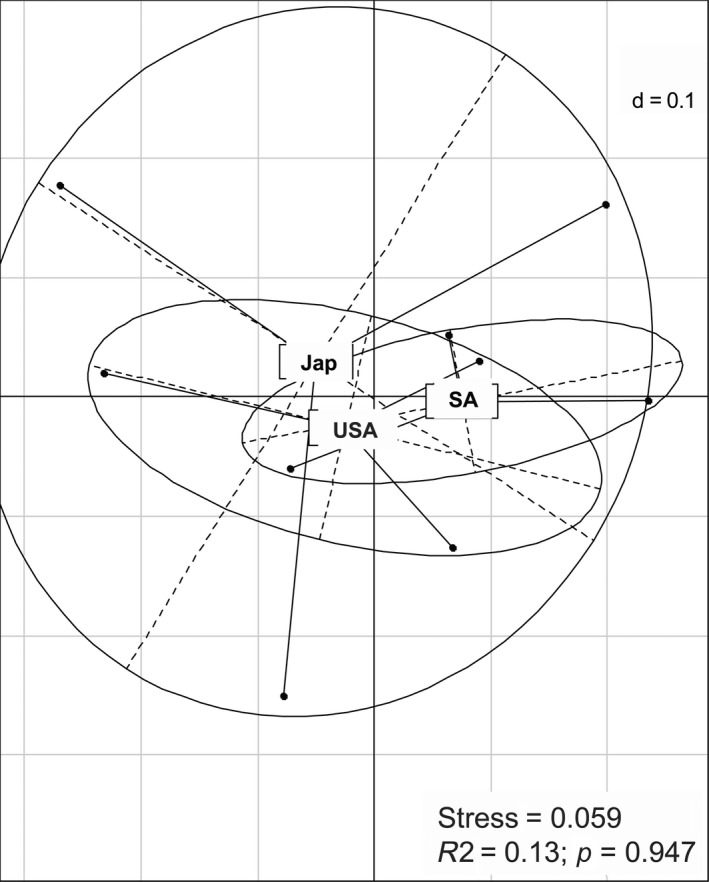
Nonmetric multidimensional scaling ordination analysis according to the presence/absence of hydrocarbons on the surface of the eggs of three populations of *Harmonia axyridis*—South Africa (SA), United States of America (USA), and Japan (Jap). There were three samples per population

Figure 1 presents the results of the nMDS analysis based on the presence/absence of ECHCs in the three populations of *H. axyridis*. The stress value is 0.059 which indicates a good fit between the reproduced distance matrix and the observed distance matrix (stress <0.1: good representation, that is, points are not placed in random positions, Clarke & Warwick, 2001). There is no significant spatial difference between the three populations studied (R^2^ = 0.13 and *p* = 0.947). The multivariate dispersion analysis shows a pattern of decrease in the dispersion of points (*F* = 3.37 and *p* = 0.1042) from Japan (0.12620) to the United States (0.08622) and finally to South Africa (0.07273).

**Figure 2:**
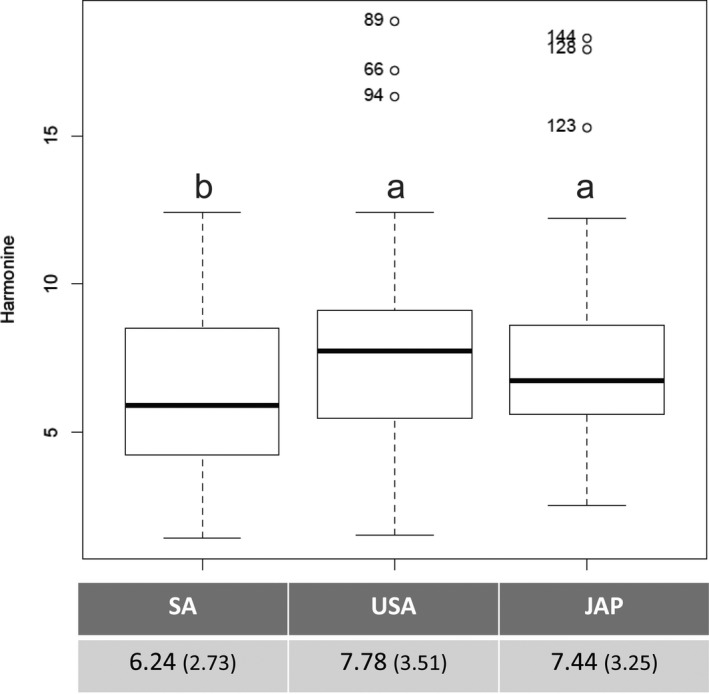
Harmonine concentration in the eggs (μg harmonine/mg of egg) of three populations of *Harmonia axyridis*—South Africa (SA), United States of America (USA), and Japan (Jap)

The concentration of harmonine in the eggs (μg harmonine/mg of egg) does not differ between the native Japanese population and the longer established population of the United States (*z* value: 0.6080; *p* = 0.5432). On the contrary, the eggs from the more recently introduced population of South Africa contain less harmonine than the eggs from both Japan and the United States (*z* value: −2.9520; *p* = 0.0036; Figure 2).

## 4 DISCUSSION

Studies on how and why non‐native populations became invasive have mostly put the emphasis on ecological explanations (Colautti & Lau, 2015; Felker‐Quinn, Schweitzer, & Bailey, 2013; Hänfling, 2007). However, contrary to what was previously believed, introduced populations either remain highly genetically diverse or are able to overcome an initial low genetic diversity (Estoup et al., 2016). Therefore, favored phenotypes can evolve allowing nonindigenous populations to spread in new environments. In the case of *H axyridis*, experiments reveal that it suffered from hardly any inbreeding depression along the invasion route and that there were improvements in several traits relating to competitive ability (Facon et al., 2011; Laugier et al., 2013). Thus, we should pay more attention to the study of key phenotypic traits that might have evolved during the invasion process of *H. axyridis* and influenced its outcome. In this study, we evaluated the evidence of evolutionary change in *H. axyridis’* chemical defenses, within the perspective of the evolution of increased competitive ability (EICA) hypothesis (Blossey & Nötzold, 1995).

It is generally accepted that the majority of insect hydrocarbons are de novo synthesized, and thus have fitness costs (Blomquist, 2010). Furthermore, harmonine, *H. axyridis* eggs’ main protection (Pell, Baverstock, Roy, Ware, & Majerus, 2008; Sloggett et al., 2011), is also of autogenous origin and is considered likely to be costly (Bezzerides, McGraw, Parker, & Husseini, 2007). Therefore, we expected that enemy release should lead to a decrease in the allocation of resources to defense in favor of increased competitive ability. Our results are consistent with this hypothesis because we found a decrease in the concentration of harmonine in the eggs and a tendency toward a decrease in the number of ECHCs along the invasion route. However, we found no differences between the three populations in the total amount of ECHCs.

In insects, cuticular hydrocarbons (CHCs) first play a role in the protection against desiccation. They are also involved in communication as contact pheromones (Blomquist & Bagnères, 2010). In ladybirds, among various communication functions (Hemptinne & Dixon, 2000), they participate in egg defense by flagging the presence of alkaloids inside the eggs (Katsanis, Magro, Ramon‐Portugal, Kenis, & Bebendreier, 2017; Ware et al., 2008).

As we found no differences in the total amount of ECHCs per egg along the invasion route, we conclude that the antidryness capacity must have been maintained in all populations. Our results might indicate that loosing antidryness capacity is costly and that ladybird eggs are relatively sensitive to desiccation, as mentioned for other insects (Gibbs & Rajpurohit, 2010; Trougakos & Margaritis, 2002). That is, trade‐offs involving the total quantity of CHCs are probably not possible.

However, we found a tendency toward a decrease in the number of ECHCs as well as changes in the relative quantities of the different compounds along the invasion route. CHCs profile variations occurred in other invasive species and are considered to depend on how narrow the initial genetic bottleneck is (Blight, Renucci, Tirard, Orgeas, & Provost, 2010; Gévar, Bagnères, Christidès, & Darrouzet, 2017; Perdereau, Dedeine, Christidès, & Bagnères, 2010). The invasive populations of *H. axyridis* experienced a bottleneck of intermediate intensity along their invasion route (Facon et al., 2011), which could explain the small differences observed. However, slight changes in CHCs profiles are enough to influence the behavior of receptors (van Zweden & d'Ettorre, 2010) and in the case of *H. axyridis* are apparently recognized by its enemies (see Rieder et al., 2008). We cannot exclude the possibility that the CHC profiles of the invasive populations have evolved in the absence of selection pressure by native natural enemies.

An unequivocal link has been shown between the harmonine content in *H. axyridis* eggs and the toxicity effects on predators (Kajita, Obrycki, Sloggett, & Haynes, 2010). Our results show that the concentration of harmonine is significantly lower in the eggs from the South African population. Heger and Jeschke (2014) and Haelewaters et al. (2017) claim that enemy release is more important during the early phases of invasion than after a long period of presence in the invaded area. This could explain why we only found differences in the concentration of harmonine of the more recently introduced South African population. In contrast to our results, Rieder et al. (2008) showed that the eggs of the *H. axyridis* from the United States were less toxic than those of the native Japanese. Their study was carried out before ours, when the USA population was in an earlier phase of the invasion process. It would be interesting to monitor the current South African and the native Japanese populations to see whether the differences in defense still exist.

A side result, that should be noted, is the fact that in our experiments, the ladybirds of the two invasive populations laid significantly larger eggs than the native Japanese ladybirds. Although we primarily measured egg weight to estimate harmonine concentration, these results add to the conclusions of Facon et al. (2011) and Laugier et al. (2013) that competitive ability is improved in invasive compared to native populations of *H. axyridis*, supporting the idea that EICA might contribute to the invasion success of the Harlequin ladybird.

Compared to plants for which it was formulated (Hänfling, 2007; Manfredini, Grozinger, & Beani, 2013), EICA has rarely been explicitly tested in animals. Furthermore, empirical evaluations of EICA have often measured growth and fecundity but only a few have assessed the differences in defensive chemistry (Asplen et al., 2012). Thus, we still have little understanding of how constitutive and induced chemical defenses vary among native and invasive populations of animals. Also, our observational work resulting from a single, nonmanipulated situation, and based on the study of a limited number of traits should be taken with caution. For a start, it would be interesting to study other *H. axyridis* invasion routes to verify our results. Moreover, this study should be extended to other life stages. Following recent works (Beckert et al., 2015; Firlej, Girard, Brehélin, Coderre, & Boivin, 2012; Schmidtberg, Röhrich, Vogel, & Vilcinskas, 2013; Verheggen, Vogel, & Vilcinskas, 2017), it would be of interest to study the evolution of immune defenses other than harmonine. At last, our study is based on the premise of enemy release. Although enemy release cannot be excluded (Ceryngier et al., 2018), a comprehensive assessment of the impact of natural enemies, including pathogens, in the native and introduced range of *H. axyridis* is still lacking (Haelewaters et al., 2017). Our common garden experiment thus corresponds to what Colautti and Lau (2015) consider to be the beginning of a conclusive demonstration of EICA, paving the way to additional experiments to identify the mechanisms underlying the evolution of the invasive populations of *H. axyridis*.

## CONFLICT OF INTEREST

None declared.

## AUTHOR CONTRIBUTIONS

A. Magro and J‐L. Hemptinne conceived and designed the work. A. Magro wrote the paper with the help of J‐L. Hemptinne; B. Facon revised the article critically. B. Facon, A. Magro. and F. Ramon‐Portugal acquired the biological material for the analyses. F. Ramon‐Portugal and C. Ducamp performed the chemical analyses. F. Ramon‐Portugal, J‐L. Hemptinne, and A. Magro did the statistical analyses.

## DATA ACCESSIBILITY

Data available from the Dryad Digital Repository: https://doi.org/https://doi.org/10.5061/dryad.df2463b
